# “Once upon a Time in the Mediterranean” Long Term Trends of Mediterranean Fisheries Resources Based on Fishers’ Traditional Ecological Knowledge

**DOI:** 10.1371/journal.pone.0119330

**Published:** 2015-03-17

**Authors:** Dimitrios Damalas, Christos D. Maravelias, Giacomo C. Osio, Francesc Maynou, Mario Sbrana, Paolo Sartor

**Affiliations:** 1 European Commission, Joint Research Centre (JRC), Institute for the Protection and Security of the Citizen (IPSC), G.03 Maritime Affairs Unit, Fisheries and Aquaculture Sector, Via E. Fermi 2749, 21027, Ispra, Italy; 2 Hellenic Centre for Marine Research (HCMR), 46.7 Km Athens-Sounio avenue, P.O Box 712, 190 13 Anavissos, Attica, Greece; 3 Institut de Ciències del Mar (ICM-CSIC), Psg. Marítim de la Barceloneta 37–49, 08003, Barcelona, Spain; 4 Consorzio per il Centro Interuniversitario di Biologia Marina ed Ecologia Applicata “G. Bacci” (CIBM), V.le N. Sauro 4 57128, Livorno, Italy; University of Vigo, SPAIN

## Abstract

We investigate long-term changes in the Mediterranean marine resources driving the trawl fisheries by analysing fishers’ perceptions (Traditional Ecological Knowledge, TEK) throughout the Mediterranean Sea during the last 80 years. To this end, we conducted an extended set of interviews with experienced fishers that enabled us to classify species (or taxa) as ‘decreasing’ or ‘increasing’ both in terms of abundance, as well as average size in the catch. The aspect that most clearly emerged in all the investigated areas over time was the notable increase of fishing capacity indicators, such as engine power and fishing depth range. Atlantic mackerel, poor cod, scorpionfishes, striped seabream, and John Dory demonstrated a decreasing trend in the fishers’ perceived abundance, while Mediterranean parrotfish, common pandora, cuttlefish, blue and red shrimp, and mullets gave indications of an increasing temporal trend. Although, as a rule, trawler captains did not report any cataclysmic changes (e.g. extinctions), when they were invited to estimate total catches, a clear decreasing pattern emerged; this being a notable finding taking into account the steep escalation of fishing efficiency during the past century. The overall deteriorating status of stocks in most Mediterranean regions calls for responsible management and design of rebuilding plans. This should include historical information accounting for past exploitation patterns that could help defining a baseline of fish abundance prior to heavy industrial fisheries exploitation.

## Introduction

The Mediterranean Sea and its environment are of great economic importance to the surrounding countries. Around 150 million people live along the shores of the Mediterranean; many coastal cities have over a million inhabitants. More notable in terms of employment (250,000 fishermen) than production (about 1.5 million tons declared in 2011 [1]), fishing is mainly on a small scale [2]. Humans have exploited the Mediterranean Sea since the prehistoric era [3]–[4]. This perennial utilization of resources has led, earlier than any other marine region of the world, to the acknowledgement that fishing may have largely affected the marine environment; manifestations in the form of protests against bottom trawling as a detrimental fishing tactic date back to 1377 A.D. and such concerns have materialized into spatio-temporal closures for fishing even since the 18th century [5], [6], [7], and prohibitions of certain fishing gears (1825 A.D. [8]). During the past two centuries, the introduction of many technological innovations has produced a progressive increase of fishing capacity, technology and catchability, a factor which further increased the pressure on the resources and the marine environment [7].

Although, there is a long history of biological research in the region [2], due to the poor condition of local economies in many countries, marine research was not a top priority. It is only recently that research has been carried out specifically in support of managing fish populations, however the level of application of research recommendations in the management of marine fisheries is still generally low. Landing trends often provide the only indication of changes that have occurred in the past. Based on a review study, 85% of the assessed stocks are currently overfished compared to a maximum sustainable yield reference point (MSY) [9]. In general, exploitation rate has been steadily increasing, selectivity (proportional exploitation of juveniles) has been deteriorating, and stocks have been shrinking [10]. However, in order to assess the true current status of the stocks, a starting point, a baseline, has to be set, so far back in time so that to coincide with the period when the stocks were at a pristine, unexploited status. It has been argued that by choosing an unsuitable baseline one will fail to properly assess the true extent of change [11]. This phenomenon, described as the “shifting baseline syndrome,” is particularly occurring in all Mediterranean fisheries assessments, given that fishermen have been trawling for at least 300 years and exploitation by other means dates back for millennia [7].

Several studies have aimed at recovering knowledge from past Mediterranean ecosystems to reconstruct ecosystem baselines [12], [13], [14]. To overcome limitations in data availability prior to the 1990’s and to recover historical information, fishers’ perceptions can be used to document changes in marine ecosystems (also called Local Ecological Knowledge, LEK, or Traditional Ecological Knowledge, TEK [15], [16], [17]). Historical memories of experienced fishermen and skippers are still vivid and can provide valuable information on decades spent at sea fishing. This approach could substantially contribute to improve the historical picture and understanding of the fisheries and associated fish communities, usually obtained from other sources. Useful information on description of fishing practices, reconstruction of the trends of exploited stocks and, changes in the species population structure can be obtained. Even though some discrepancies due to emotionality of the past memories can sometimes confine the information, its added value is indubitable to reconstruct a past picture of marine environment. Disqualifying such information as “anecdotal” dismisses important first-hand information. In addition, fishermen’s traditional ecological knowledge (TEK) could have an important role in the management process; this kind of information could constitute a complementary source to integrate new knowledge in fisheries biology and marine ecology.

In this study we explored fishers’ perceptions throughout the northern Mediterranean Sea in the most extensive study conducted to date (26 fishing ports, 91 old fishers interviewed). The same group of fishers has recently provided valuable information regarding the dramatic historical decline of Mediterranean elasmobranchs and marine mammals [18]. This time our focus was on the commercial species driving the bottom trawl fisheries from the past up till now, investigating how their relative abundance and average size has evolved through time. By doing so we were able to classify species (or taxa) as ‘decreasing’ or ‘increasing’ both in terms of abundance, as well as average size in the catch. The plausible drivers behind these trends were further considered.

## Materials and Methods

### Fishers’ perception

Information was gathered by means of questionnaire-based interviews following a standardized sampling protocol [15], [19]. No ethics statement was required by the funding agency (European Commission, Directorate General for Maritime Affairs & Fisheries—EC, DG MARE). All interviewed fishers (during the DG MARE Tender ‘EVOMED’) collaborated on a voluntary basis. An ‘Oral Consent process’ was followed: participants were provided all of the necessary information on the nature of the study before obtaining their consent. ‘Oral consent’ is sufficient for this type of studies since no personal sensitive data is planned to be disclosed. To this end the datasets made available to PLoSONE, use masked codes (for the fields: Country, Area, Period, Species) and lack actual names of persons, making it impossible to track the individual fishers.

**Fig 1:**
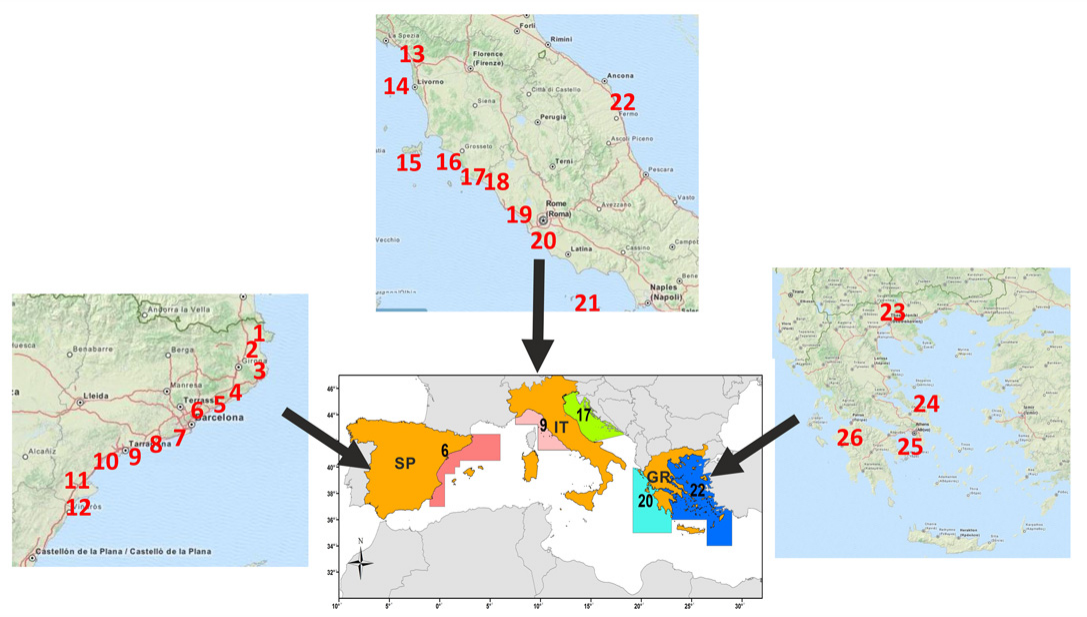
Map showing the ports where the interviews with the fishermen were carried out. SPAIN (GSA 6): 1: Port de la Selva; 2: Roses; 3: Palamos; 4: Blanes; 5: Arenys de Mar; 6: Mataro; 7: Barcelona; 8: Vilanova i la Geltrù; 9: Tarragona; 10: Cambrils; 11: L’Ametlla de Mar; 12: San Carles de la Rapita; ITALY (GSA 9 & 17): 13: Viareggio; 14: Livorno; 15: Elba Island; 16: Castiglione della Pescaia; 17: Porto Santo Stefano; 18: Porto Ercole; 19: Civitavecchia; 20: Fiumicino; 21: Ponza Island; 22: Civitanova Marche; GREECE (GSA 20 & 22) 23: Nea Michaniona; 24: Chalkis; 25: Peireas; 26: Patra. Country maps source: ©OpenStreetMap contributors. http://www.openstreetmap.org/copyright

A total of 91 experienced fishermen in 26 fishing ports belonging to five FAO GFCM (Food and Agriculture Organization of the United Nations—General Fisheries Commission for the Mediterranean) Geographical Sub-Areas (GSAs) of the northern Mediterranean Sea were interviewed (Fig. 1): Catalan Sea (GSA 6) in Spain; Ligurian, northern-central Tyrrhenian Sea (GSA 9) and northern Adriatic Sea (GSA 17) in Italy; Ionian (GSA 20) and Aegean Sea (GSA 22) in Greece. Fishermen were between 45 and 88 years old (median 70). One of them has entered the fishery in the distant past (1932) with the median year of starting activity being 1955; no fisher in our sample had less than 27 years of experience in the field. Thus, as a result of the interviewees’ longtime experience, a coverage of almost 80 years of observations was obtained.

The questionnaire was designed to study the perception of the oldest active and retired fishers on the evolution of bottom trawling, principally targeting demersal fish and occasionally small pelagics. Interviews were carried out during 2009 and 2010 in selected ports of Spain, Italy and Greece. The main contents of the questionnaire concerned information about vessels, fishing gears, fishing practices and fishing grounds (location of main fishing grounds, duration of fishing trips, on board activity, etc.), the main target species, estimation of catches (the usual catch, memories of exceptional captures, sizes of specimen caught, etc) and discard compositions. The choice of sites/areas where the interviews were collected was based on: (a) the importance of the local fisheries, at a national level, in terms of total production, employment and number of fishermen; (b) the historical aspect: existence of experienced skippers; (c) an already existing mutual confidentiality and respectful relationship between the fishing associations/individual fishermen and the researchers; (d) constraints such as time and distance. In order to facilitate the estimation of eventual changes over time, questionnaires were designed to collect information during three main time periods: 1940 to 1959, 1960 to 1979, and from 1980 to present. Upon recording fisherman’s age, vessel characteristics and fishing tactics, the local fish names mentioned were linked to taxonomic ones. Only species brought up during the interview by the fisherman were noted down. Respondents were asked to qualitatively rank chronological abundance of these taxa, by assigning one of the following scores: ‘*Much more abundant*’, ‘*More abundant*’, ‘*The same*’, ‘*Less abundant*’, and ‘*Not able to evaluate*’. Change in size of valuable commercial species was assessed by classifying average size by time period into one of the following: ‘*Larger*’, ‘*More or less the same*’, ‘*Smaller*’, and ‘*Not able to evaluate*’. By combining responses of each respondent over all time periods, the recorded taxa were assigned to a trend factor: species showing a decreasing trend (“D”), species showing an increasing trend (“I”) and species not showing any noticeable trend (classified as stable or “S”). Additionally to the aforementioned qualitative information, quantitative estimates of total catch rates were recorded, expressed in kg (or boxes) landed per fishing day. All information gathered concerned species caught in the bottom trawl fisheries. Details on the protocol followed and the structure of the questionnaires are available in [20] and as supplementary material (S1 File).

### Statistical analyses

All taxa mentioned in each interview were structured in a presence/absence matrix, interviews being the samples (rows) and taxa the variables (columns). Interview data was accompanied by the corresponding information: identified Trend, Country, Geographical Area, Time Period, Fishing Depth and, Vessel characteristics. Each ‘interview x time period’ was considered as an independent replicate sample. To investigate differences in the temporal trends of each taxon among regions, the non-parametric multivariate permutational analysis of variance (Permanova) was employed [21]. Whereas ordinary ANOVA/MANOVA assumes normal distributions and implicitly Euclidean distance, Permanova can work with any distance measure that is appropriate to the data using permutations to make it distribution-assumption free. A two way Permanova based on Bray-Curtis resemblance matrix was used to test for the terms “Country” (with 3 levels: Spain, Italy, Greece) and “Trend” (with 3 levels: ‘“D-Decrease”, “I-Increase”, “S-Stable”’), both considered as fixed crossed factors. Whenever Permanova results were significant, the Similarity Percentages Procedure (SIMPER) was employed [22] to identify taxa that were most important in each region regarding abundance/size trends. A Non-metric Multi Dimensional Scaling (nMDS) ordination was performed to visualize geographical patterns in abundance/size trends. Only taxa observed three or more times, in the whole series of interviews, were considered. All the multivariate analyses were performed in R library *vegan* (Community Ecology Package ver. 2.0–10).

Assuming that non-linearities are most likely to occur in the functional relationships between catch estimates and explanatory variables, catch rates were modelled as a function of six factors (*Period*, *Country*, *GSA*, *Depth*, engine power-*kW* and *Fisherman*) by applying Generalized Additive Mixed Models (GAMM) techniques. GAMMs are extensions of Generalized Additive Models (GAMs [23]) with some of the predictors being treated as random variables. The fact that the same fishers’ responses are used induces correlations among the observations, which are important for the model to capture. One very effective way of achieving this is to attribute a random effect to each response, treating *Fisherman* as a random effect, by implying a GAMM. The use of GAMMs in fisheries science, although not widely used, is gaining recognition [24]. To model catch rates (total catch/fishing day), we used the function *bam* (*mgcv* package in R [25]) as it allows specification of random term smoothers, specification of weights, offset and it can handle large datasets while still providing good estimates when there are few random effects levels. Plausible combinations among predictor variables, plus interactions among them, would have generated a sizeable number of candidate models. To avoid investigation of numerous irrelevant models, we focused on those combinations linked to the problem under study [26]. As a result a series of empirical candidate models were constructed including meaningful combinations of the six parameters under investigation that plausibly influenced catch rates (Table 1). ‘Best’ model selection was based on a comparison of the AIC-GCV (Akaike Information Criterion—Generalized Cross Validation) statistical scores [25]. Comparisons via GCV score and via AIC normally yield similar answers [27], and AIC was used. Once the ‘best’ model was identified, parameters were estimated using REML (REsidual Maximum Likelihood) statistical score since it gives better parameter estimates [28]. All the analyses were performed in R v.3.0.3 [29]. Additionally to the standard approach which standardizes the trends but does not account for technological improvement (also known as ‘technological creeping’), we reconstructed technological improvement on Mediterranean trawl vessels based on a recently applied methodology [7]. Time scaled correction coefficients were used as an offset parameter, representing the increase in trawl efficiency. 1.00 was selected over the period 1940–1959 (considering catching power constant), 1.72 from 1960 to 1979 and 2.24 from 1980 to 2008.

**Table 1:** The set of candidate models.

*MODEL*	*Linear Predictor*
**m1**	s(kW:Period) + s(Depth: Period) + Period + Country+ s(Fisherman, bs = “re”)
**m2**	s(kW: Period) + s(Depth: Period) +Period + Country + GSA +s(Fisherman, bs = “re”)
**m3**	s(kW: Period) + s(Depth) + Period + Country + GSA + s(Fisherman, bs = “re”)
**m4**	s(kW) + s(Depth) + Period + Country + GSA +s(Fisherman, bs = “re”)
**m5**	s(kW: Period) + Depth + Period+ Country + GSA +s(Fisherman, bs = “re”)
**m6**	s(kW: Period) + Depth + Period+ Country + s(Fisherman, bs = “re”)
**m7**	s(kW: Period) + Depth + Period+ GSA + s(Fisherman, bs = “re”)
**m8**	s(kW: Period) +Period+ Country + s(Fisherman, bs = “re”)
**m9**	s(kW: Period) + Country + s(Fisherman, bs = “re”)

GSA = Geographical Sub-Areas

s() is a smooth function represented using penalized regression splines [25].

Covariate “Fisherman” was estimated through penalized random effects (bs = “re”).

## Results

**Fig 2:**
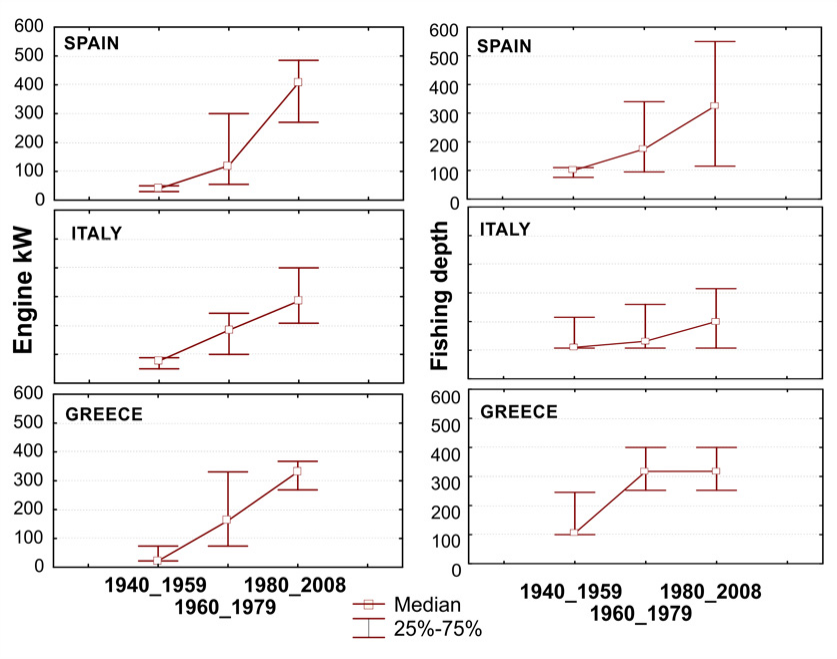
Median overall engine power (in kW) and fishing depth (in meters) of the vessels used by the fishermen interviewed over time. Upper and lower whiskers indicate 25–75% percentiles around the median.

Temporal representation of the fleet operational characteristics illustrates how fishing capacity amplified at a fast pace; fishing activities expanded from coastal/shelf areas to distant deeper waters. While in the past the median fishing depth was shallower than 100 m, in the recent years working depths have exceeded 300 m; obviously promoted by the immense escalation of engine power (Fig. 2), allowing skippers to explore new unexploited fishing grounds.

The assemblage of responses included a total of 42 species (or taxa) (Table 2). 14 species/taxa were mentioned more than 10 times; the three dominating species being: European hake (*M*. *merluccius*), red mullet (*M*. *barbatus*) and deep-water rose shrimp (*P*. *longirostris*). These species were brought up by fishers in more than 1/3 of the interviews.

**Table 2:** List of taxa reported by the fishers.

Taxon	English name	Abundance			Size trend		
		D	S	I	D	S	I
*Aristeus antennatus*	Blue and red shrimp	4	0	2	1	4	1
*Aristaeomorpha foliacea*	Giant rd shrimp	0	0	1	0	2	0
*Boops boops*	Bogue	6	4	1	2	10	0
*Citharus linguatula*	Spotted flounder	1	0	1	1	1	0
*Dicentrarchus labrax*	Sea bass	0	1	1	0	2	0
*Engraulis encrasicolus*	Anchovy	6	2	1	1	7	1
*Eledone spp*.	Horned/musky octopus	8	6	4	3	12	2
*Illex coindetii*	Broadtail shortfin squid	1	1	0	1	1	0
*Lithognathus mormyrus*	Striped seabream	3	1	0	0	3	0
*Loligo spp*.	Squids	3	5	1	0	7	1
*Lophius spp*.	Anglerfishes	2	3	1	1	5	0
*Mullus barbatus*	Red mullet	15	21	5	9	32	1
*Merluccius merluccius*	European hake	38	27	11	17	56	6
*Micromesistius poutassou*	Blue whiting	10	5	3	5	12	2
*Mullus surmuletus*	Striped red mullet	3	8	1	0	11	0
*Mullus spp*	Red mullets	8	3	7	5	10	4
*Nephrops norvegicus*	Norway lobster	11	9	4	2	21	2
*Octopus vulgaris*	Common octopus	5	3	2	2	6	1
*Pagellus bogaraveo*	Blackspot seabream	0	0	1	1	0	0
*Pagellus erythrinus*	Common Pandora	1	1	1	1	1	1
*Penaeus kerathurus*	Caramote prawn	2	2	1	1	3	1
*Parapenaeus longirostris*	Deep sea pink shrimp	10	21	5	4	28	1
*Pomatomus saltator*	Blue fish	0	1	0	0	1	0
*Raja spp*.	Rays	2	0	0	0	2	0
*Sparus aurata*	Gilthead seabream	1	1	0	1	1	0
*Scyliorhinus canicula*	Small spotted catshark	0	0	1	0	1	0
*Sparisoma cretense*	Mediterranean parrotfish	1	2	4	0	7	0
*Spicara flexuosa*	Picarel	0	2	0	0	2	0
*Squilla mantis*	Mantis shrimp	6	6	3	1	12	1
*Sepia officinalis*	Cuttlefish	4	0	2	1	6	0
*Sardina pilchardus*	Sardine	1	2	1	0	2	2
*Scomber scomber*	European mackerel	10	1	1	2	10	0
*Spicara smaris*	Picarel	5	5	1	2	10	0
*Solea vulgaris*	Common sole	3	0	0	1	2	0
Scophthalmidae	Flatfishes	0	1	0	0	1	0
*Scorpaena spp*.	Scorpionfishes	5	2	0	2	3	0
*Squalus spp*.	Squalid sharks	0	1	0	1	0	0
*Trisopterus minutus capelanus*	Poor cod	5	0	1	3	3	0
*Trachurus trachurus*	Horse mackerel	0	2	0	0	1	0
*Trachurus spp*.	Horse Mackerels	4	6	2	1	11	1
Triglidae	Gurnards	1	2	0	0	1	0
*Zeus faber*	John Dory	2	1	0	0	3	0

The number of times in which they were assigned to the groups ‘INCREASE’ (‘I’), ‘DECREASE’ (‘D’) and ‘STABLE’ (‘S’) is reported.

### Abundance trends

**Fig 3:**
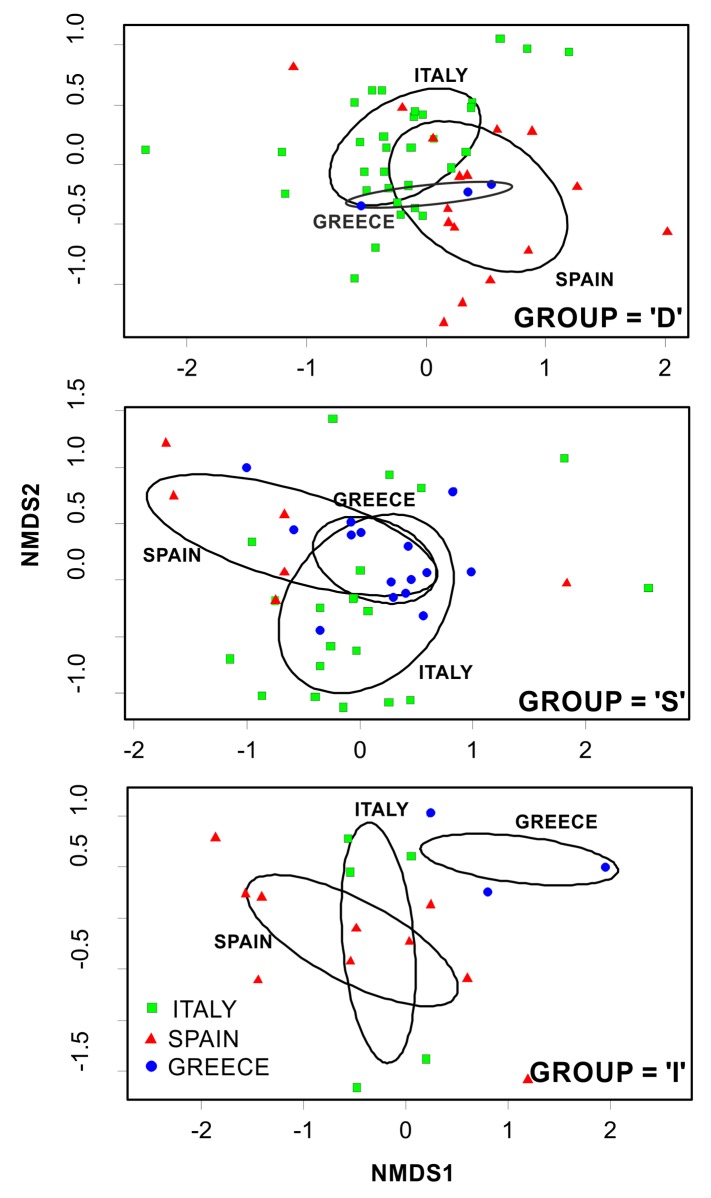
Non-metric Multi Dimensional Scaling (nMDS) ordination comparing species abundance trends responses outputs across the different locations (Country). The position of each dot is defined by the assemblage of species recorded in each interview.

Most species/taxa were classified under all three trend categories, by the different fishers, making it difficult to infer a straightforward conclusion on their status (Table 2). However, Permanova analysis highlighted significant differences by geographical criteria (Country) and time period (Trend) (Table 3 top). The Non-metric Multi Dimensional Scaling (nMDS) ordinations demonstrated the geographical structure of the observed trends in the datasets (“D”, “I”, “S”) (Fig. 3). SIMPER analyses (Table 4) identified the species/taxa contributing mostly into each group and indicated a quite high separation among groups (overall between groups dissimilarities: ‘I’ vs ‘D’ 81.34%; ‘D’ vs ‘S’ 79.07%; ‘S’ vs‘I’ 82.66%).

**Table 3:** Results of the PERMANOVA Analyses.

ABUNDANCE	Df	SumsOfSqs	MeanSqs	*F*.Model	*R* ^2^	Pr(>*F*)
*Trend*	2	1.574	0.78694	2.6392	0.03727	0.01
*Country*	2	4.290	2.14509	7.1940	0.10160	0.01
*Trend*: *Country*	4	1.772	0.44307	1.4859	0.04197	0.04
*Residuals*	116	34.588	0.29818	0.8191		
*Total*	124	42.225	1			

SIZE	Df	SumsOfSqs	MeanSqs	*F*.Model	*R* ^2^	Pr(>*F*)
*Trend*	2	0.8962	0.44808	1.7804	0.02934	0.05
*Country*	2	4.1434	2.07172	8.2319	0.13564	0.01
*Trend*: *Country*	3	0.8445	0.28151	1.1186	0.02765	0.37
*Residuals*	98	24.6636	0.25167	0.8073		
*Total*	105	30.5478	1			

Permutational multivariate analyses of variance based on the Euclidean dissimilarity measure for presence-absence data. The tests were done using 9999 permutations under the reduced model.

**Table 4:** Abundance trends: most important taxa characterizing groups ‘I’,‘D’ and‘S’ by SIMPER analysis.

GROUP	Taxon	Average frequency of occurrence	Contribution (%)	Cumulative Sum (%)
**D**	*T*. *min*. *capelanus*	0.200	5.62	5.62
	*S*. *scomber*	0.290	5.33	10.95
	*L*. *mormyrus*	0.128	5.17	16.12
	*Scorpaena spp*.	0.151	4.86	20.98
	*Z*. *faber*	0.081	4.65	25.63
**S**	*M*. *surmuletus*	0.227	6.47	6.47
	*Loligo spp*.	0.116	5.64	12.11
	*S*. *pilchardus*	0.050	5.2	17.31
	*Lophius spp*.	0.062	5.17	22.48
	*Trachurus spp*.	0.089	5.09	27.57
**I**	*S*. *cretense*	0.583	10.45	10.45
	*P*. *erythrinus*	0.154	6.94	17.39
	*S*. *officinalis*	0.220	6.76	24.15
	*A*. *antennatus*	0.220	6.76	30.91
	*Mullus spp*.	0.537	6.46	37.37

The fish taxa are listed in decreasing order of their importance in typifying the groups ‘INCREASE’ (‘I’), ‘DECREASE’ (‘D’) and‘STABLE’ (‘S’) by SIMPER analysis performed on presence/absence data. Cut off for low contributions: 90.00%. ‘I’ vs‘D’ overall between group dissimilarity 81.34; ‘D’ vs‘S’ overall between group dissimilarity 79.07; ‘S’ vs‘I’ overall between group dissimilarity 82.66

### Decreasing (“D”)

Fifty-eight percent of the fishers listed one or more species/taxon as decreasing (Table 2). In total, 33 species/taxa were listed. Of those most frequently listed the most common were valuable commercial species such as European hake, red mullet, deep-water rose shrimp, and Norway lobster. Other commercial species followed, such as horned and musky octopuses, blue whiting, mantis shrimp, striped red mullets and various species of mackerels. It must be noted here, that these absolute numbers of species occurrence cannot directly typify the abundance trend group; this is realized through SIMPER analyses. Looking at the “D” nMDS ordination plot (Fig. 3 top), there is a partial overlap among all regions (Spanish, Italian, Greek). According to SIMPER analysis (Table 4), the first 5 species contributing mostly in characterizing group “D” were: poor cod, Atlantic mackerel, striped seabream, scorpionfishes, and John Dory.

### Stable (“S”)

Fifty-three percent of the fishers considered, listed one or more species/taxon as not being affected in terms of abundance throughout the years (Table 2); 33 species/taxa in total were listed. European hake, red mullet, and deep-water rose shrimp were the most common ones. The “S” nMDS ordination plot (Fig. 3 mid), demonstrated an almost complete overlap between Greek and Italian fishers responses and a reasonable separation between Italian and Spanish fishermen perceptions. According to SIMPER analysis (Table 4), the species contributing mostly in characterizing group “S” were: striped red mullet, Loligo squids, sardine, anglerfish, and horse mackerels.

### Increasing (“I”)

A minor fraction of fishers, twenty-three percent, listed one or more species/taxon as increasing (Table 2). In total, 29 species/taxa were listed. From the “I” nMDS ordination plot (Fig. 3 bottom), there was a complete separation between the eastern region (Greece) and the central-west Mediterranean (Italy, Spain) as well as a significant overlap between Spain and Italy. According to SIMPER analysis (Table 4), the first 5 species contributing in characterizing group “I”, were: Mediterranean parrotfish, common pandora, cuttlefish, blue and red shrimp, and mullets.

### Size trends

**Fig 4:**
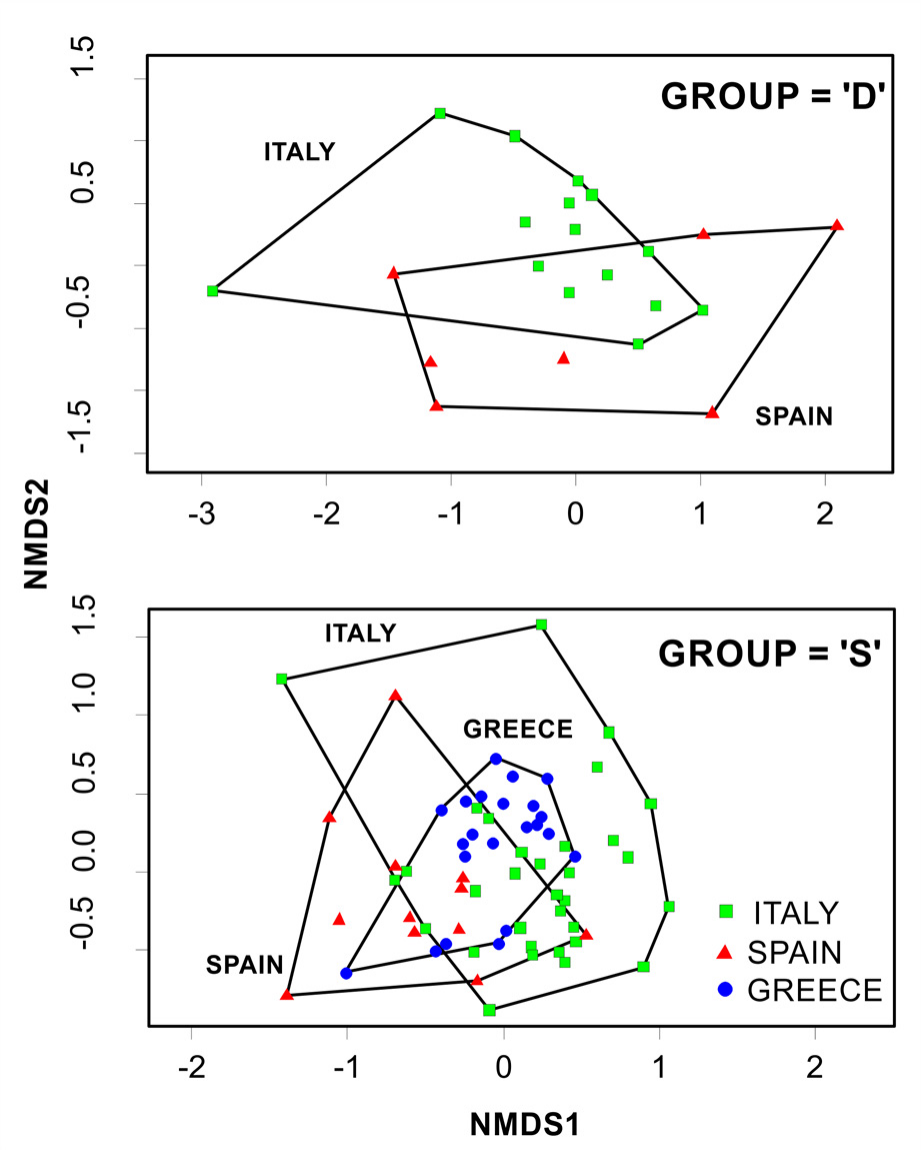
Non-metric Multi Dimensional Scaling (nMDS) ordination comparing species size trends responses outputs across the different locations (Country). The position of each dot is defined by the assemblage of species recorded in each interview.

Several species/taxa were classified among all three trend categories (Table 2). Permanova analysis highlighted significant differences by Country and time Period (Table 3 bottom). The Non-metric Multi Dimensional Scaling (nMDS) ordinations demonstrated the geographical structure of the observed trends in the datasets (“D” and “S”) (Fig. 4). Group “D” did not account for Greece, since only one Greek fisher suggested a decrease in average size; group “I” was not plotted, since only few Spanish fishermen (and one Italian) observed an increasing size trend. In comparison to abundance trends, SIMPER analyses indicated a lower level of separation among groups (overall between group dissimilarities: ‘D’ vs‘S’ 73.81%; ‘I’ vs‘D’ 76.85%; ‘S’ vs‘I’ 76.96%).

### Decreasing (“D”)

Only twenty-seven percent of interviewed fishers mentioned one or more species/taxon as decreasing in size (Table 2). In total, 27 species/taxa were listed. Looking at the “D” nMDS ordination plot (Fig. 4 top), there is a separation between the Spanish and Italian region (with a partial overlap). SIMPER analysis (Table 5) revealed the species contributing mostly in characterizing group “D”, and having undergone a significant decrease in their average size: poor cod, various scorpionfishes, common sole, common pandora and European hake.

**Table 5:** Size trends: most important taxa characterizing groups ‘I’,‘D’ and‘S’ by SIMPER analysis.

GROUP	Taxon	Average frequency of occurrence	Contribution (%)	Cumulative Sum (%)
**D**	*T*. *min*. *capelanus*	0.273	10.01	10.01
	*Scorpaena spp*.	0.154	8.29	18.30
	*S*. *vulgaris*	0.070	7.07	25.37
	*P*. *erythrinus*	0.070	7.07	32.44
	*M*. *merluccius*	0.149	5.80	38.24
**S**	*S*. *cretense*	0.125	4.71	4.71
	*M*. *surmuletus*	0.160	4.60	9.31
	*Loligo spp*.	0.082	4.16	13.47
	*S*. *officinalis*	0.069	4.10	17.57
	*S*. *mantis*	0.102	4.01	21.58
**I**	*S*. *pilchardus*	0.964	18.83	18.83
	*P*. *erythrinus*	0.497	14.21	33.04
	*P*. *kerathurus*	0.300	8.69	41.73
	*A*. *antennatus*	0.235	7.32	49.05
	*Mullus spp*.	0.688	6.64	55.69

The fish taxa are listed in decreasing order of their importance in typifying the groups ‘INCREASE’ (‘I’), ‘DECREASE’ (‘D’) and‘STABLE’ (‘S’) by SIMPER analysis performed on presence/absence data. Cut off for low contributions: 90.00%. ‘I’ vs‘D’ overall between group dissimilarity 76.85; ‘D’ vs‘S’ overall between group dissimilarity 73.81; ‘S’ vs‘I’ overall between group dissimilarity 76.96

### Stable (“S”)

A large part of the interviewees (78%) listed one or more species/taxon as not having experienced any change in body size; almost all species/taxa were listed (40) (Table 2). The “S” nMDS ordination plot (Fig. 4 bottom), demonstrated an almost complete overlap between Greek and Italian fishers responses and a moderate separation between Italian and Spanish fishermen perceptions. According to SIMPER analysis (Table 5), the species contributing mostly in characterizing group “S” were: Mediterranean parrotfish, striped red mullet, Loligo squids, cuttlefish and mantis shrimp.

### Increasing (“I”)

Only one out of ten fishers, listed one or more species/taxon as increasing in body size. In total, 16 species/taxa were listed (Table 2). According to SIMPER analysis (Table 5), the species contributing more than 55% in characterizing group “I”, were: sardine, common pandora, caramote prawn, blue and red shrimp, and red mullets.

### Total catch rates trends

Assuming a normal distribution for the underlying dataset, we examined a series of candidate models based on their AIC scores. Model *m*_*1*_ was selected as the most suitable (Table 6):

$$f\left(E\left[Catch\right]\right)=c+a_{1}Country+a_{2}Period+s_{1}\left(kW:Period\right)+s_{2}\left(Depth:Period\right)+s_{3}(Fisherman)$$

**Table 6:** Generalized Additive Mixed Model results for factors affecting total catches.

		df	F	P-value
**Parametric Terms**	as.factor(Period)	3	46.453	<2e-16
	as.factor(Country)	2	0.027	0.974

		edf	F	P-value
**Approximate significance of smooth terms**	s(kW):Period1940_1959	4.226	3.897	0.0031
	s(kW):Period1960_1979	5.368	2.343	0.0351
	s(kW):Period1980_2008	1.000	6.228	0.0141
	s(avg.depth):Period1940_1959	4.400	3.850	0.0026
	s(avg.depth):Period1960_1979	2.692	2.346	0.0718
	s(avg.depth):Period1980_2008	1.636	0.586	0.5541
	s(Fisherman):dum1	62.836	2.836	< 2e-16
**R-sq.(adj)**	0.728			
**Deviance explained**	85.2%			
**REML score**	1278.1			
**Scale est.**	30314			
**n**	190			

s: smooth function represented using penalized regression splines

df: degrees of freedom

edf: estimated degrees of freedom

F: F-ratio test score

P-value: refers to the p-values from an ANOVA F-ratio test

“:”: interaction among terms

R-sq: The adjusted r-squared for the model. Defined as the proportion of variance explained

REML: Random efects maximum likelihood score

Family: gaussian; Link function: identity; Formula (Response variable as a function of predictor variables): catch_kg ~as.factor(Period) + s(kW, by = Period) + s(avg.depth, by = Period) + as.factor(Country) + s(Fisherman, bs = “re”, by = dummy var).

**Fig 5:**
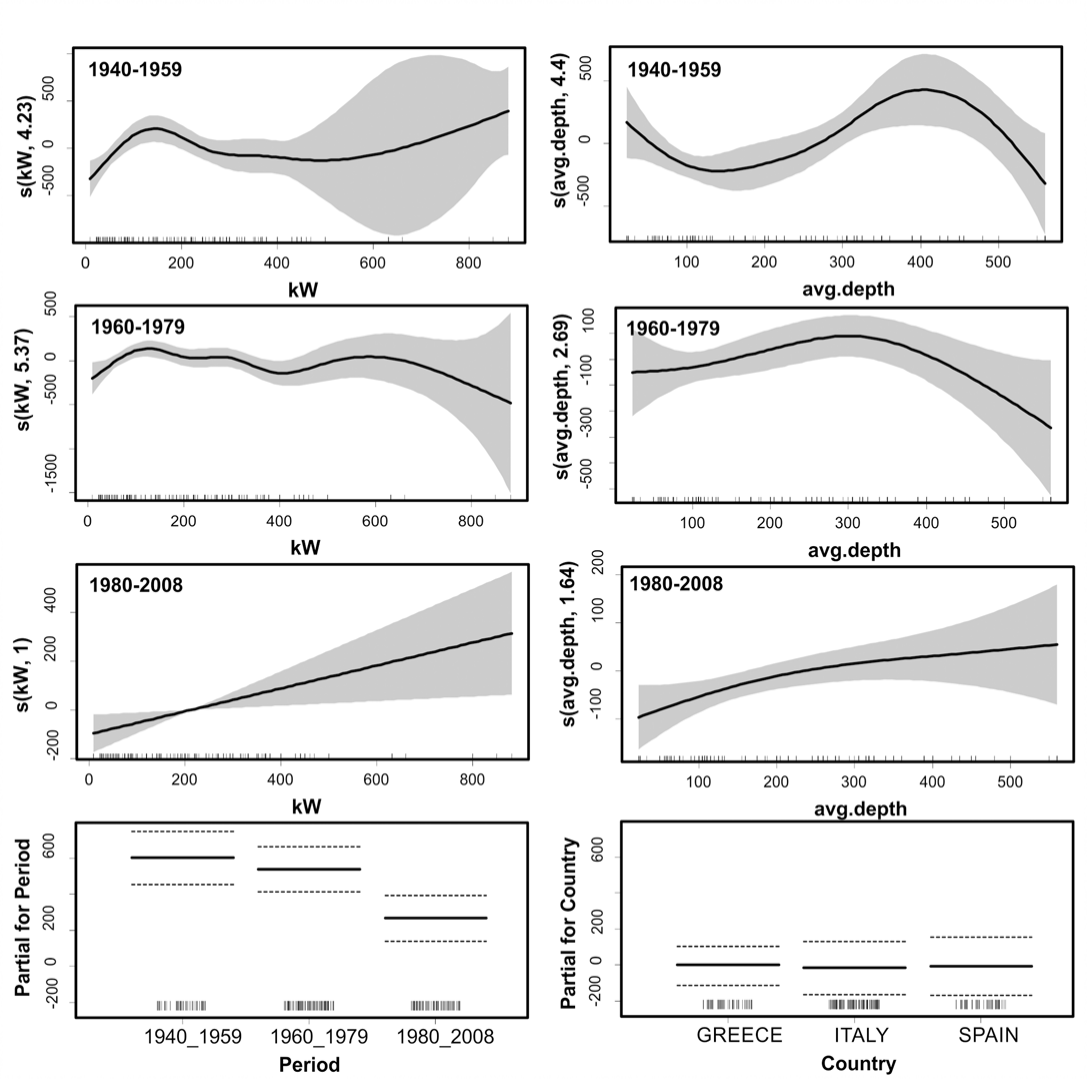
Generalized additive mixed model (GAMM) derived effects of engine power (kW), fishing depth, Period, and Country on the catch rates reported by fishers. Gray shaded area and dashed lines of upper and lower brackets indicate 2 standard errors above and below the estimates shown in solid lines. The relative density of data points is shown by the ‘rug’ on the x-axis.

**Fig 6:**
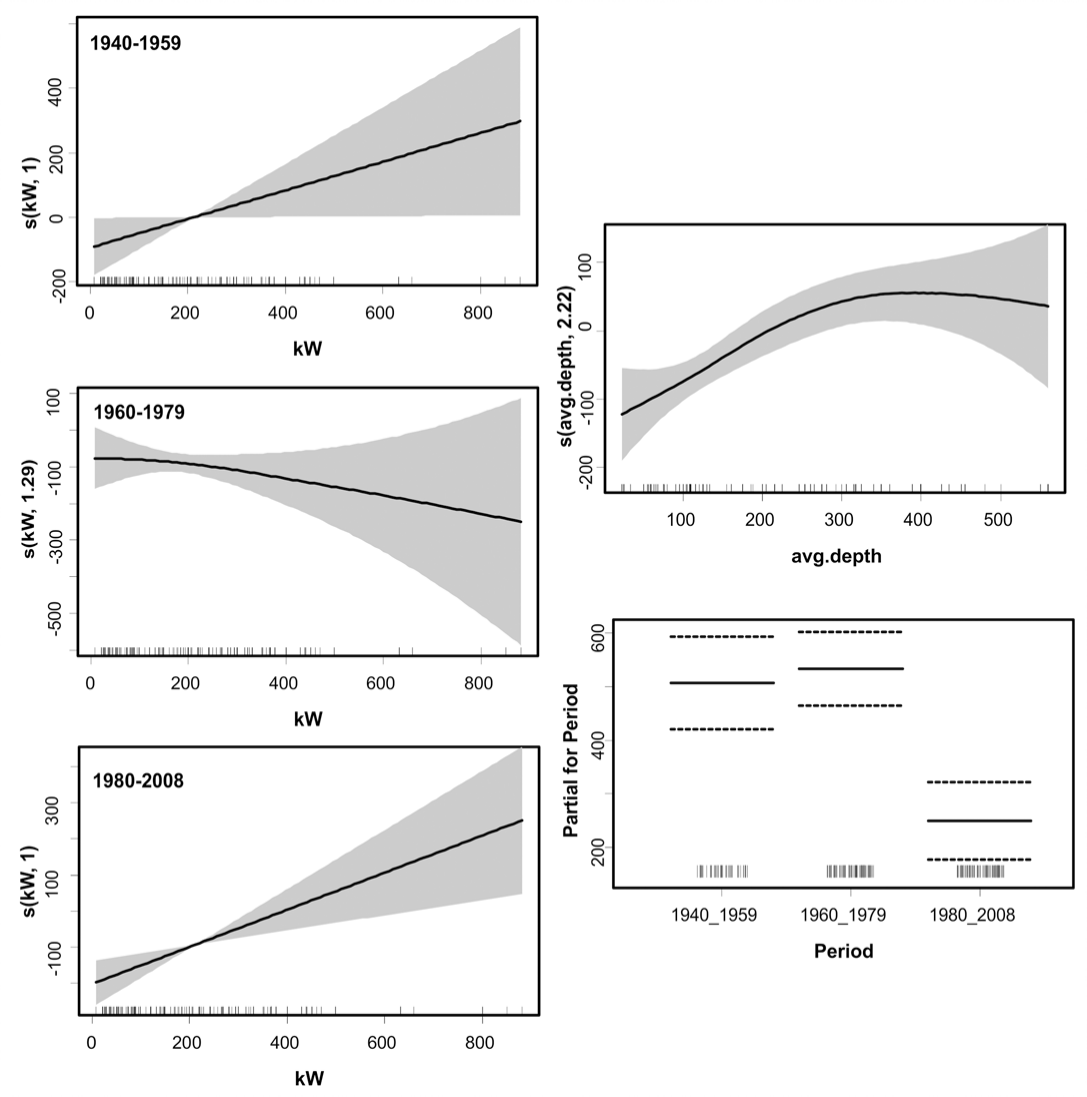
Generalized additive mixed model (GAMM) derived effects of engine power (kW), fishing depth, and Period on the catch rates reported by fishers, after correcting for technological creeping. Gray shaded area and dashed lines of upper and lower brackets indicate 2 standard errors above and below the estimates shown in solid lines. The relative density of data points is shown by the ‘rug’ on the x-axis.

The model explained a considerable percentage (85.2) of the variance in the perceived catch rates, the main explanatory variables being: interactions of *Period* with *kW* and *Depth*, and *Fisherman* (which was treated as a random effect). The likelihood of a larger catch was higher during the early years; in the most recent period catch estimates were significantly lower than those before the 1980’s (Fig. 5). Fishing depth and engine power (kW) effects were weak during the two early periods, however during the last period (post 1980) it seems that increased horse power and deep water fishing became significant drivers of elevated catch rates. Furthermore, when taking into account the correction factor for technological creeping throughout the years, this decreasing temporal catch trend was once more confirmed (Table 7 and Fig. 6).

**Table 7:** Generalized Additive Mixed Model results for factors affecting total catches, taking into account correction for technological creeping.

		df	F	P-value
**Parametric Terms**	as.factor(Period)	2	34.29	1.87e-12

		edf	F	P-value
**Approximate significance of smooth terms**	s(kW):Period1940_1959	1.002	4.162	0.0434
	s(kW):Period1960_1979	1.294	0.830	0.3877
	s(kW):Period1980_2008	1.000	3.915	0.0501
	s(avg.depth)	2.217	2.357	0.0830
	s(Fisherman):dum1	64.127	2.974	< 2e-16
**R-sq.(adj)**	0.659			
**Deviance explained**	78.8%			
**REML score**	1299.9			
**Scale est.**	37912			
**n**	190			

s: smooth function represented using penalized regression splines

df: degrees of freedom

edf: estimated degrees of freedom

F: F-ratio test score

P-value: refers to the p-values from an ANOVA F-ratio test

“:”: interaction among terms

R-sq: The adjusted r-squared for the model. Defined as the proportion of variance explained

REML: Random efects maximum likelihood score

Family: gaussian; Link function: identity; Formula (Response variable as a function of predictor variables): catch_kg ~ s(kW, by = Period) + s(avg.depth) + Period + offset(Q) + s(Fisherman, bs = “re”, by = dummy var).

## Discussion

The interviews performed during this study constituted the first attempt to collect information in such a way on a wide geographical scale in the Mediterranean. In general, the fishermen demonstrated interest and wide availability to provide information on their past activities. As a rule, fishermen were pleased to see scientists looking over their past experiences and actually wondered why the decision-making centres such as ministries, government and the European Commission have never seriously considered their views.

Many fisheries scientists are still rather sceptical against fishers’ historical information and tend to disqualify it as ‘anecdotal’, seeing history as ‘unscientific’. Traditional ecological knowledge (TEK) or ‘anecdotes’ (as fisheries scientists call it), represents a very important source of knowledge falling under an entire specialty in history known as oral history [30]–[31]. Sáenz—Arroyo et al. [15] elegantly argue how (and why) fisheries science and marine conservation biology can be perceived as an historical science, in a similar way as evolutionary biology is. They stress that, in order to correctly explain the patterns and processes prevailing today, we need to consider how human impacts have affected wild population in the past applying not only methods traditionally used by fisheries scientists, but also employing methods used by historians.

During this study, the aspect that most clearly emerged in all the investigated areas was the notable increase of fishing capacity indicators over time, such as engine power and fishing depth range. Horse power was undoubtedly the parameter showing the highest temporal increase: the present values are, on average, five-to-eight times higher, than those of the earlier periods. The vessels used at present are notably different in terms of size and technological equipment compared to those employed in the past, providing also higher working standards. Technological innovations have improved the operational capacity of the fleets allowing them to abandon their traditional shallow coastal fishing grounds and start visiting distant deeper waters, fully exploiting the water column, a pattern in accordance with a general expansion of fishing activities documented worldwide [32], [33], [34].

### Abundance trends

In spite of many Mediterranean Institutions being involved since the late 19th century in the field of fishery science, knowledge on the historical evolution of the exploited populations is still rather scarce and limited to restricted areas and time periods. This aspect hampered, for most of the commercial species, the investigation of the temporal evolution of abundance. Only after the early 1990’s, through the introduction of the MEDITS international trawl survey [35] and since 2002, with the implementation of a regular data collection for all EU Mediterranean waters [36]–[37], the EU Mediterranean fisheries are regularly monitored. As a result, knowledge on the status of the resources is largely based on the relatively good quantitative information of the past twenty years [9]–[38], however most stock assessments rely on data from the past 10 years.

Based on the same set of interviews, fishermen reported a striking decline for elasmobranch species and marine mammals throughout the Mediterranean [18]; a pattern largely supported by recent studies [39], [40], [41]. Atlantic mackerel (*Scomber scomber*), poor cod (*Trisopterus minutus capelanus*) and John Dory (*Zeus faber*) were identified as species showing a significant decline in abundance. In general, fish of the Scombridae family, are considered a heavily fished group all over the world and the Atlantic populations are considered to be in a decreasing trend over the past 20 years [42]. Coinciding with this time frame, in the Mediterranean, It has been suggested [13] that late 1990’s were the point in time at which *Scomber scomber* status downturned from ‘abundant’ to ‘occasional’ in catches. This was attributed to the negative impacts of confounding effects such as: expanding fisheries, habitat degradation and pollution. Poor cod has been classified as ‘overexploited’ in the most recent assessment of Mediterranean stocks [43]. It is recommended that current poor cod fishing mortality rates should be reduced by 18% if exploitation has to be in line with sustainable levels of fishing. An evident decline in abundance of John Dory has been reported in the Adriatic Sea, based on comparisons of historical bottom trawl surveys (‘Hvar’ 1948–1949) with concurrent ones (MEDITS) [44]–[45]. The biological characteristics of this species, mainly the late age and size at maturity [46] make it one of the most sensitive species to fishing pressure.

On the other hand, a limited number of species were perceived as demonstrating an increasing abundance trend. Among them, the Mediterranean parrotfish (*Sparisoma cretense*), a species of thermophilic nature of the southern Mediterranean. It has been argued [13] that it belongs to the list of organisms considered to be good indicators of changes associated with warming in the marine environment. Mediterranean fisheries are threatened by a shift in the climate regime, the main feature of the current period being the increased variability in the observed phenomena. These changes have a faster effect on the comparatively small and semi-enclosed Mediterranean Sea than on the world ocean [47]. The recorded changes in temperature and rainfalls, among others, are associated to dramatic changes in Mediterranean biota. In the last 50 years, enhanced by both the opening of the Suez Canal, aquaculture and ship transport, hundreds of non indigenous species reached and established themselves in the Mediterranean Sea basin. The majority of them are of warm-water affinity. In parallel, it might be expected that higher temperature represents “climate deterioration” for the indigenous species of coldwater affinity. Poor cod‘s decreasing trend may also be related to the warming tendency of Mediterranean waters. Poor cod’s preference for cooler water masses within the continental shelf of the eastern Mediterranean has been documented [48]. In contrast, blue and red shrimp (*Aristeus antennatus*) observed increase was most likely a masked effect of the gradual expansion of the fleets’ fishing depth range, targeting unexploited virgin populations residing in the deep. This species, also known as the deep-sea shrimp, has a eurybathic distribution in the Mediterranean, and although it colonizes several habitats between 100 and 3000m of depth, an increased abundance at the deeper layers (1400–1500m) has been identified [49].

### Size trends

Temporal changes in the size of the specimen caught were less noticeable than changes in abundance. The cases of poor cod (*Trisopterus minutus capelanus*) and scorpionfishes (*Scorpaena spp*.) need to be highlighted, since both of them exhibited a simultaneous decrease in abundance and size. Taking into account that average mesh size of trawl net has increased from less than 20 mm in the 1940s up to more than 40 mm currently [20], it is alarming that average body size of captured fish kept decreasing. It is documented that substantial improvements in size selectivity of commercially important species should be achieved by switching from smaller to larger mesh cod-ends [50]–[51]. Moreover, one of the most common commercial species driving Mediterranean demersal fisheries, the European hake (*Merluccius merluccius*), was among the list of ‘shrinking’ fish. These findings were also corroborated by the results of the MEDITS survey running since 1994. Annual trends in the lengths of the large fish and specifically the average 95^th^ percentile of total length distribution (average total length of the large fish making up less than 5% of the sample), give clear signs of populations lacking large individuals, both for European hake and poor cod, since the late 1990’s. In addition, during the 50’s in the North Tyrrhenian Sea [52]–[53], a much larger average size for hake has been reported, compared to the one observed in the current trawl surveys. ‘Shrinking’ of fishes has been contended for a long time, not denying it is an observable fact, but mostly debating on who/what is to hold responsible for. In a most recent review [54] plausible causes are summarized to: (i) direct removals of oldest and largest individuals through size-selective harvesting, (ii) contemporary evolution towards smaller size-at-age owing to selective harvesting of fast growing individuals or climate change, (iii) contemporary evolution towards increased/earlier energy allocation to reproduction, and consequently smaller realized size-at-age owing to high fishing mortality, and (iv) physiological declines in growth rates owing to increasing temperatures and decreasing oxygen concentration in warmer oceans. Some seem to be convinced that climate change is the main driver behind changes in fish size structure. It has been suggested [55] that averaged maximum body weight is expected to shrink by 14–24% globally from 2000 to 2050 under a high-emission scenario; tropical and intermediate latitudinal areas being the most heavily impacted. In few cases, results were so compelling to exclude environmental factors as the underlying cause of the observed declines, and clearly identify overfishing as the prime agent (NW Atlantic shelf fish communities [56]).

### Total catch rates

When trawler captains were invited to estimate total overall catches, a clear decreasing pattern emerged. In almost all areas, production during the ′40-′60 period was significantly higher than the one observed recently. This is quite a remarkable finding taking into account how efficient have the modern fishing fleets become, compared to the ones in the past. Elevated efficiency can be straightforwardly interpreted as an increase in catchability. The recent project EVOMED [20] described in detail how fishing capacity evolved and technological improvements occurred over time in the Mediterranean fisheries, notably affecting fishing power and catch efficiency (estimated to increase by 1–2% annually).

It is likely that the perception on the amount of catch over time, may have been biased by several factors, such as the different discard rate throughout the years (in the past discards were significantly less) and the different gear selectivity. In a broad sense, fishermen observe a non-standardized catch rate that is largely shaped by unnoticed changes over time: new fishing grounds, alterations in gear, innovative fishing tactics, altered target species and markets. The result may be a misleading perception in the change in abundance. However, by taking into account the increasing fishing efficiency we aimed to correct for any misconceptions and hopefully grasped a valid change in resource relative abundance, which was conclusively decreasing. Concurring with these findings, a recent study on historical catches from the Hellenic Seas stressed that recent levels of relative abundance for certain demersal species, were estimated as low as just 20% of the initial ones back in the 60s [57]. The most extensive search of historical data sources and publications carried out in the Mediterranean [7], concludes that the drop in biomass in most regions was extremely large; highest relative biomass identified back in the 1920s with a second lower peak in the 1960s and contemporary biomass even lower. The further back the series was reconstructed, the larger the decline in demersal biomass. These findings were realized as a quantification of the shifting baseline syndrome: today we are assessing stocks solely based on data from the past 20 years, which correspond to the recent part of the historical trends, having no knowledge of the decline’s extent. The overall deteriorating status of demersal stocks in most Mediterranean regions [9]–[10] should trigger multidisciplinary and multilateral collaborations to design a feasible management scheme. Ecosystem based and regionalized management by means of multiannual plans are among the most important innovative aspects of the reform of the European Common Fishery Policy [58]. Incorporation of historical information could assist proper assessment by accounting for exploitation history as well as for constructing rebuilding plans.

## Supporting Information

S1 File EVOMED protocols. (PDF)
