# Supplementary material for: “Once upon a Time in the Mediterranean” Long Term Trends of Mediterranean Fisheries Resources Based on Fishers’ Traditional Ecological Knowledge
Source: PLoS One. 2015 Mar 17;10(3):e0119330. doi: 10.1371/journal.pone.0119330 (PMC4364015; doi:10.1371/journal.pone.0119330)
Supplement: S1 File — (PDF) [file pone.0119330.s001.pdf]

CHAPTER 3

WP3 - Historical fishermen knowledge

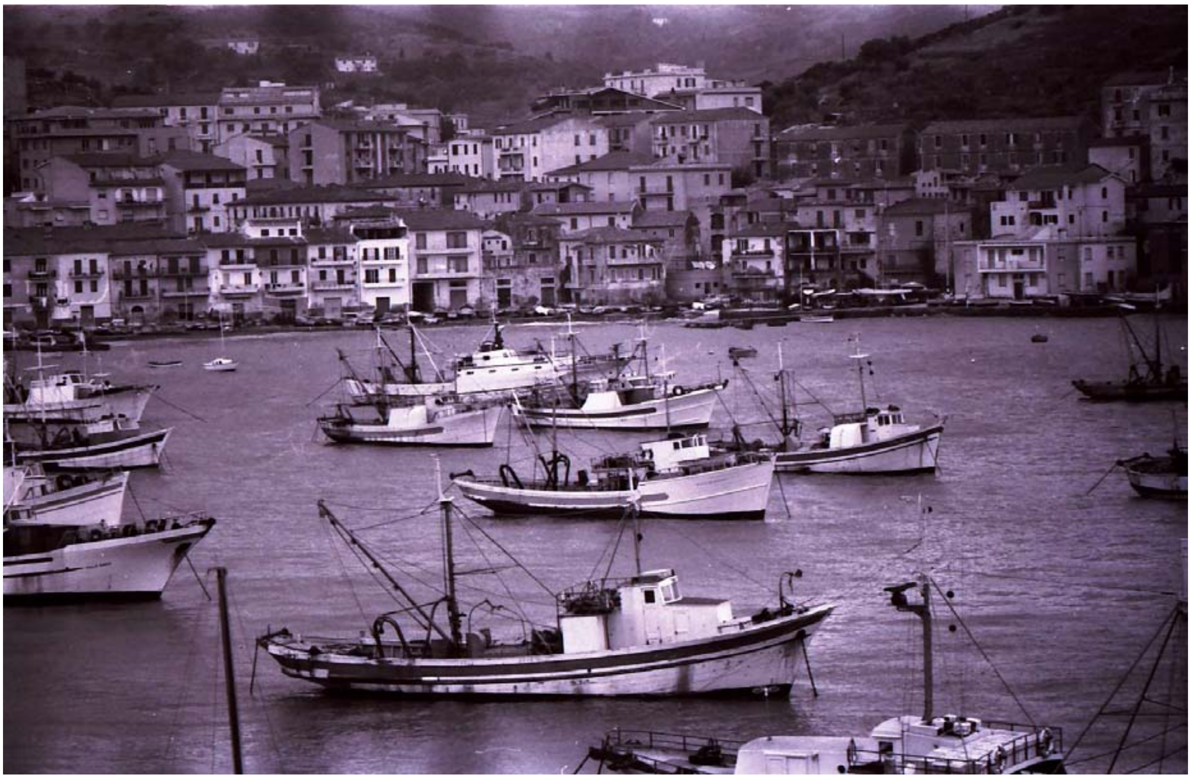

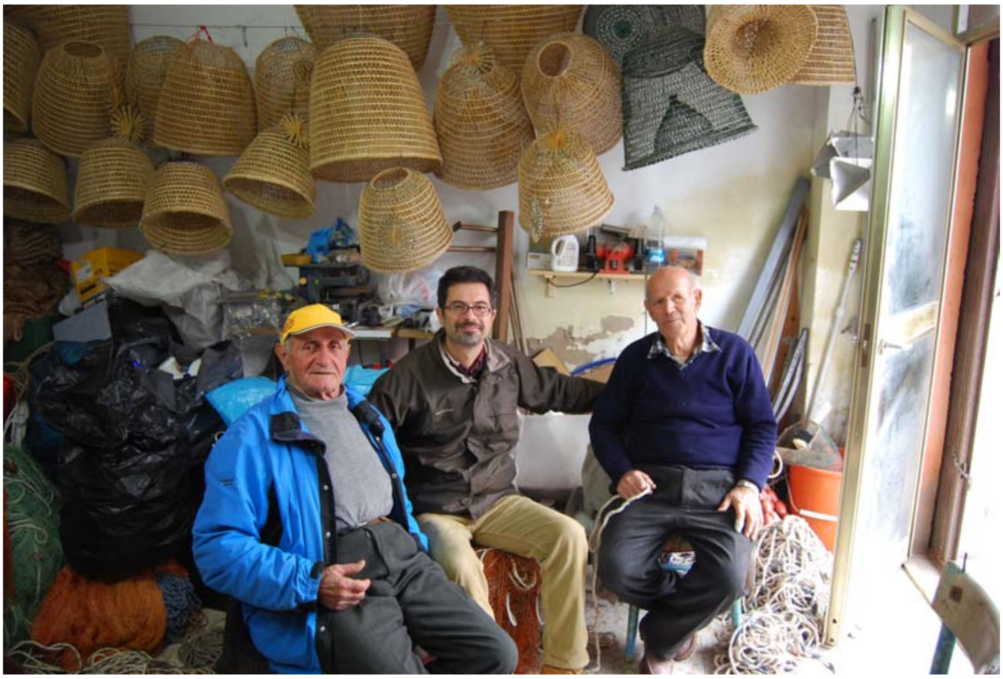

3.1 - Introduction

An important aspect to take into account in the research of historical data is to gather information from old fishermen and fishing captains. Their historical memories on past activities are still vivid and can provide valuable information on decades spent at sea fishing.
This approach could substantially contribute to improve the historical picture and understanding of the fisheries and associated fish communities obtained from the other sources. Useful information for a better description of fishing practices, for the reconstruction of the trends of exploited stocks, changes in the species catch composition can be obtained. Even though some discrepancies due to emotionality and affectivity of the past memories can sometimes confound the information, its added value is indubitable to reconstruct a past picture of marine environment. Disqualifying such information as "anecdotal" dismisses important first-hand information. Historical fishermen knowledge will be available for not much longer as the fishing captains that participated to the fishing expansion in the 1950's are now getting very old.
In addition, fishermen's traditional ecological knowledge (TEK) could have an important role in the management process; this kind of information should constitute a complementary source to get new knowledge in fisheries biology and marine ecology.
For these reasons, it was decided to create a specific workpackage (WP3) of EVOMED for the realization of interviews to fishermen by all the partners involved in this project. Particular effort was spent in this activity which, although very time consuming and in spite of the difficulties in extracting quantitative data and standardise them, provided interesting results.
In this context, the main objective of WP3 was to gather and summarise information coming from the historic fishermen knowledge, in order to obtain additional data on fishing practices, and species caught in the past decades. The collected information will be used to integrate the picture of the evolution of Mediterranean fisheries in the 20th Century, obtained from fishery dependent and independent sources.

3.2 - Methods

In the first months of the project the activities were focused on organizing the work and defining the methodological aspects of this WP. A large part of the first coordination meeting was dedicated to these aspects.
A standardised sampling protocol, evaluated also by the social scientist Garcia Allut, was defined during the first months of the project and applied by all the partners. The approach was similar to that adopted in the past in similar studies carried out in other areas (Bergmann *et al*., 2004; Sáenz-Arroyo *et al*., 2005), consisting to carry out interviews on a significant sample of fishermen using a pre-defined questionnaire. Other methods, like Delphi method or Estimate-talk-Estimate (ETE), result not adequate to the scope of WP3. They are normally applied when the number of interviewed persons is very low, for example, when there is the necessity to agree on solutions to issues among a group of stakeholders.
In order to facilitate the approach to fishermen (older active fishermen and retired fishermen) and to contact the highest number possible of people, several strategies have been followed.
First of all, contacts with fishermen and fishermen associations were established in the sites or ports where the EVOMED partners work, in order to inform about the interviews and to spread the word about the upcoming interviews, to gather the highest number possible of fishermen.
In many cases, the interviewing process started by first contacting via telephone the single person (if already known) or a responsible of the local Fishermens' Association to obtain names and contact details of retired fishermen (asking specifically for persons involved in trawl fishing and preferably skippers). In several occasions the EVOMED partners had already well-established

relations with fishers that facilitated the whole procedure. Still a variety of responses were encountered: most of them reacted positively in the idea of being interviewed, a few also provided further contacts of retired fishers but others declined.
Moreover, a leaflet was prepared, describing the objectives and finalities of the interviews (**see Annex 3.1**); it was widely distributed in the coastal places chosen for this activity. Publicity of the EVOMED project and of the fishermen interviews was made also by the web sites of the Institutions involved in this project.
The choice of sites/areas where the interviews were collected was based on:
a) the importance of the local trawl fisheries, at a national level, in terms of total production, employment and number of fishermen;
b) the historical, aspect i.e. it was necessary for EVOMED to collect a representative sample of the oldest active and retired trawl fishermen;
c) the areas selected as case study for this project;
d) an already existing mutual confidentiality and respectful relationship between the fishing associations/individual fishermen and the researchers;
e) other constraints such as time and distance.

At the same time, following the guidelines set during the first coordination meeting, the questionnaire for the interviews was prepared (**Deliverable 3.1, see Annex 3.2).** In order to standardise the information coming from the interviews, a common standardised format was adopted to register data coming from different sites.
The main contents of the questionnaire concerned information about vessels, fishing gears, fishing practices and fishing grounds (location of main fishing grounds, duration of fishing trips, on board activity, etc.), the main target species, estimation of catches (the usual catch, memories of exceptional captures, sizes of specimen caught, etc) and discard compositions. Particular attention was given to information on species composition of all the exploited fauna (including not commercial species). The questionnaire contains a specific section dedicated to species of particular interest, such as marine mammals and selachians. For this last group the questionnaire is focused on collecting information on catches, sightings, economic importance and disappearance of species from the catches over time.
The questionnaire was planned to collect information according to three main time periods, in order to facilitate the estimation of eventual changes over time: from 1940's to 1960's and from 1960's to 1980's of the last Century, and from 1980's of the last Century to present. The interview protocol has been constructed to maximise the reliability of the information gathered and to obtain as much as possible quantitative information.
In mid-October the interviews started in the selected areas: Catalan Sea (Spain), Ligurian, northern and central Tyrrhenian Seas (Italy), northern Adriatic (Italy), Aegean Sea (Greece), Ionian Sea (Greece). In the selection of the fishermen to interview, preference was given to vessel Captains.
Most information concerns trawl fisheries, only a smaller part concerns small scale fisheries (mostly trammel nets and gill nets); however, all the information collected focused on demersal resources.

At the same time of the realization of the interviews, a common database in Excel was prepared in a standard format to store all the information gathered (**Deliverable 3.2**). The structure of the database strictly reflects that of the questionnaire; it has been prepared according to four different sheets: "fishermen interview"; "vessel-gear-trip"; "catch-marketing"; "fishes-of-particular-interest".
The data entry has been planned according to a fixed codification, in order to minimize the errors of data input. Tab. 3.2.1 summarizes the structure of the database.
The completed database with all the collected information is provided as an Annex to the present report, in a cd-rom (**Deliverable 3.4**).
In order to guarantee the privacy, all the possible sensitive data of the fishermen interviewed (as name and surname, name of the vessels, personal information), were excluded from the database.

All data gathered with the interviews were analysed in order to quantify the information collected. Summary reports (**Deliverable 3.5**) have been produced for the following case studies:

1) Trawl fishery in the Catalan Sea (GSA6)
2) Trawl fishery in the Ligurian Sea, northern and central Tyrrhenian sea (GSA9)
3) Small scale fishery in the Ligurian Sea, northern and central Tyrrhenian sea (GSA9)
4) Trawl fishery in the central Adriatic sea (GSA17)
5) Trawl fishery in the Greek Ionian and Aegean seas (GSA 20 and 22)

Tab. 3.2.1 – Structure of the Excel database prepared to store the information collected through the fishermen interviews.

**Sheet**<br>**Fishermen interview**	**Sheet**<br>**Vessel_gear_trip**	**Sheet**<br>**Catch_Marketing**	**Sheet**<br>**Fishes-of-particular-interest**
**Fields:**<br><br>N. of Interview<br>Date<br>Place<br>Tape recorded<br>File code<br>Age<br>Place of origin<br>Ports of activity<br>Starting activity (year)<br>Education 1<br>Education 2<br>Education 3<br>Education 4<br>Education 5<br>Notes interviewer<br>Photos<br>Articles<br>Captain logbooks<br>Films<br>Video-rec. interview	**Fields:**<br><br>N. of Interview<br>**Period**<br>Mooring port<br>Overall length (m)<br>Tonnage<br>Radar (year)<br>GPS (year)<br>Sonar (year)<br>Radio VHF (year)<br>Echo-sounder (year)<br>Other<br>Engine kW<br>Gear Type	**Fields:**<br><br>N. of Interview<br>**Period**<br>Past catches compared with those of the present<br>Most abundant species 1<br>Most abundant species 2<br>Most abundant species 3<br>Most abundant species 4<br>Sizes of target species compared with the present<br>Examples<br>Unit of the Catch x Effort<br>Catch<br>Minimum catch<br>Maximum catch<br>Presence of benthic organisms<br>Examples<br>Main Comm. sp. discarded 1<br>Main Comm. sp. discarded 2<br>Main Comm. sp. discarded 3<br>Main Comm. Sp. discarded 4<br>Reasons of discarding<br>Period of max pres. juveniles<br>Examples<br>More econ. relevant sp. 1<br>More econ. relevant sp. 2<br>More econ. relevant sp. 3<br>More econ. relevant sp. 4<br>More econ. relevant sp. 5	**Fields:**<br><br>N. of Interview<br>**Period**<br>Dolphin Incidental Catches<br>Whale Incidental Catches<br>Seal Incidental Catches<br>Turtle Incidental Catches<br>Dolphin Intentional Catches<br>Whale Intentional Catches<br>Seal Intentional Catches<br>Turtle Intentional Catches<br>Gear<br>Purpose<br>Dolphin Sighting Frequency<br>Whale Sighting Frequency<br>Seal Sighting frequency<br>Turtle Sighting frequency<br>Elasmob. catches compared with the present<br>% Elasmob. on the Total Catch<br>Elasmob. main species caught 1<br>Elasmob. main species caught 2<br>Elasmob. main species caught 3<br>Elasmob. main species caught 4<br>Disapp. Elasmobranch species 1<br>Disapp. Elasmobranch species 2<br>Disapp. Elasmobranch species 3<br>Disapp. Elasmobranch species 4<br>Last catch of disapp. species

3. 3 - Results

This chapter reports the results of the interviews carried out with the fishermen in different places of the Mediterranean. Overall, 107 interviews were carried out (**Deliverable 3.3**), collecting a highly diversified information as concerns fishing grounds exploited and fishing patterns. The majority of them concern trawl fisheries, only a few were collected for small scale fisheries mainly using set nets. The duration of each individual interview ranged on average from 1 to 2 hours.
The interviews were carried out in 20 ports of Spain, Italy and Greece (Fig. 3.3.1).
The collected information has allowed producing the following five summary reports (**Deliverable 3.5)**:

3.3.1 - Trawl fishery in the Catalan Sea (GSA6)
3.3.2 - Trawl fishery in the Ligurian Sea, northern and central Tyrrhenian Sea (GSA9)
3.3.3 - Small scale fishery in the Ligurian Sea, northern and central Tyrrhenian Sea (GSA9)
3.3.4 - Trawl fishery in the central Adriatic Sea (GSA17)
3.3.5 - Trawl fishery in the Greek Ionian and Aegean Sea (GSA 20 and 22)

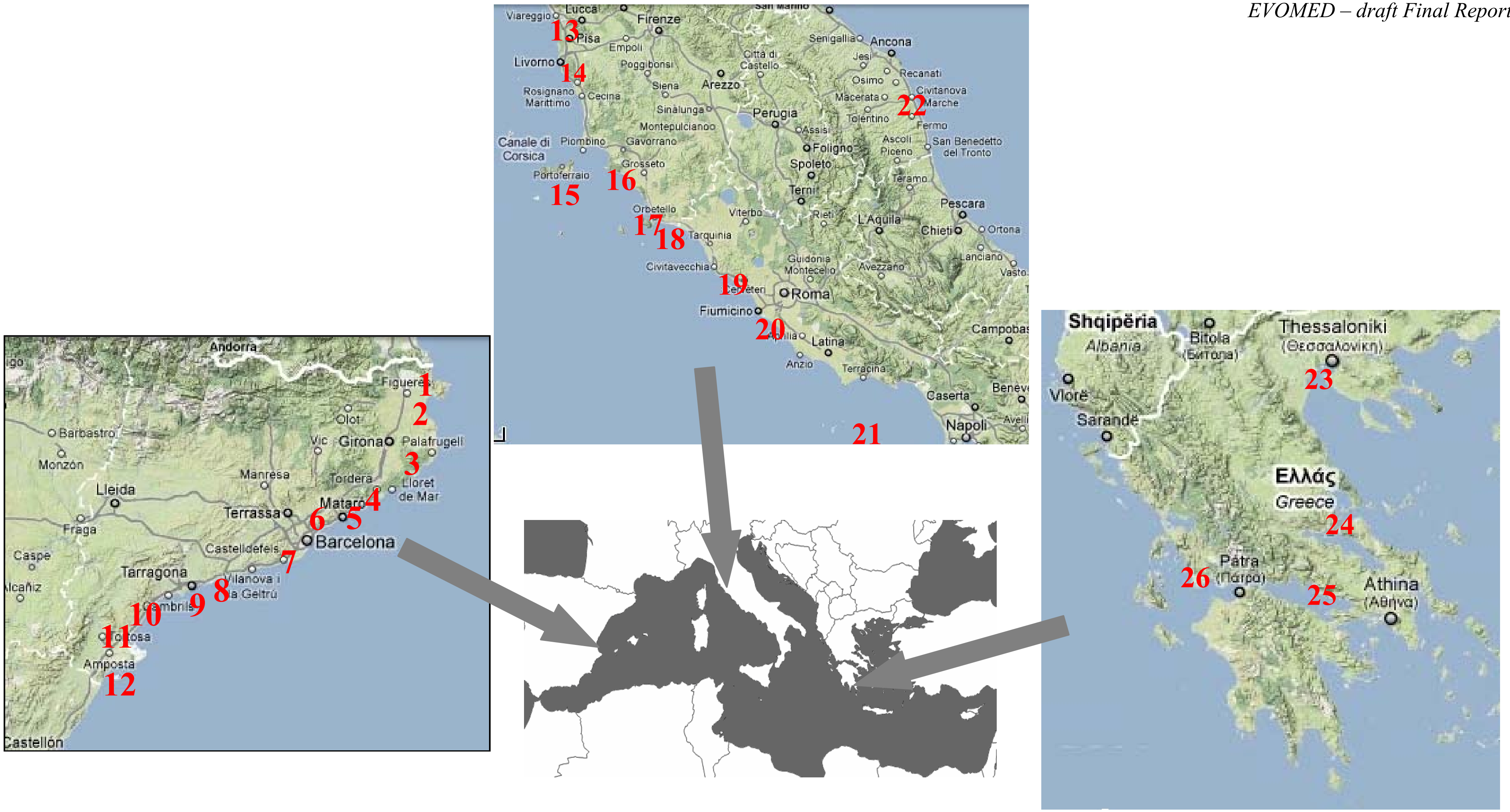

Fig. 3.3.1 - Map showing the ports where the interviews with the fishermen were carried out.
1: Port de la Selva; 2: Roses; 3: Palamos; 4: Blanes; 5: Arenys de Mar; 6: Mataro; 7: Barcelona; 8: Vilanova i la Geltrù; 9: Tarragona; 10: Cambrils; 11: L'Ametlla de Mar; 12: San Carles de la Rapita; 13: Viareggio; 14: Livorno; 15: Elba Island; 16: Castiglione della Pescaia; 17: Porto Santo Stefano; 18: Porto Ercole; 19: Civitavecchia; 20: Fiumicino; 21: Ponza Island; 22: Civitanova Marche; 23: Nea Michaniona; 24: Chalkis; 25: Peireas; 26: Patra.

ANNEX 3.1

INVITATION TO ALL THE "OLD FISHERMEN"

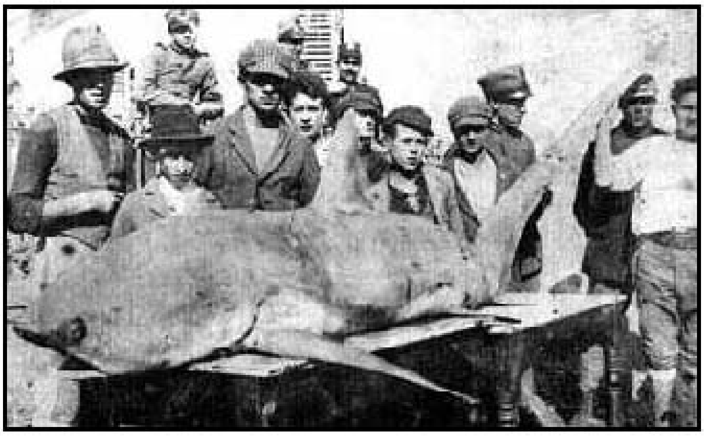

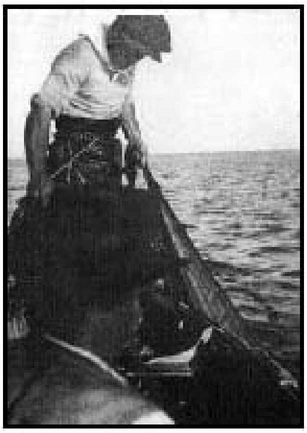

FROM AUGUST 2009 IS ACTIVE A PROJECT OF THE EUROPEAN COMMUNITY CALLED EVOMED TARGETED TO COLLECT INFORMATION OF THE PAST CENTURY ON MEDITERRANEAN FISHING ACTIVITY. THE INFORMATION OF MOST INTEREST ARE:

- *FISHNIG GEARS OF THE PAST*
- *VESSELS USED*
- *CATCHES AND LANDINGS*
- *HISTORIES OF PAST FISHING TRIPS*

... AS WELL AS OTHER CURIOSTIES AND ANECDOTES USEFUL TO RECONSTRUCT THE HISTORY OF MEDITERRANEAN FISHERIES FROM AFTER SECOND WORLD WAR TO NOWDAYS. WE INVITE, THEREFORE, ALL THE "OLD" FISHERMEN FOR A SMALL TALK.

CONTACT US, WE WILL COME TO YOU!

CONTACTS:

Paolo Sartor and Mario Sbrana

Tel. e Fax 0586 260723

E-mail: psartor@cibm.it

ANNEX 3.2

EVOMED
(EU Contract n° SI2.539097)

Fishermen interview

N° interview:		
Date:	Place:	
Interviewers:		
Tape Recorder:	☐ YES	☐ NO
File code:		

Name/forename interwieved	
Age	
Place of origin	
Ports of activity	
Starting activity (year)	
Education	1)
	2)
	3)
	4)
	5)

**VESSEL DESCRIPTION**			
	From 40's to 60's	From 60's to 80's	From 80's to present
1) Name of the vessel			
2) Mooring port			
3) Overall length (m)			
4) Tonnage (GRT and/or GT)			
5) Navigation instruments	Radar<br>from year:	Radar<br>from year:	Radar<br>from year:
	gps<br>from year:	gps<br>from year:	Gps<br>from year:
	Sonar<br>from year:	Sonar<br>from year:	Sonar<br>from year:
	Radio (VHF)<br>from year:	Radio (VHF)<br>from year:	Radio (VHF)<br>from year:
	Echo-sounder<br>from year:	Echo-sounder<br>from year:	Echo-sounder<br>from year:
	Other	Other	Other
6) Number of persons embarked			
7) Role of the interviewed			
8) Fish conservation	Ice house<br>from year:	Ice house<br>from year:	Ice house<br>from year:
	Ice production<br>from year:	Ice production<br>from year:	Ice production<br>from year:
	Boxes material	Boxes	Boxes
	Other	Other	Other

**ENGINE**			
	From 40's to 60's	From 60's to 80's	From 80's to present
9) kW/Hp<br>Real or nominal?			

MAIN GEAR			
	From 40's to 60's	From 60's to 80's	From 80's to present
10) Type (local name)			
11) Length and width of the net (number of meshes)			
12) Vertical opening (m)			
13) Mesh size (cod end) (mm)			
14) Net material			
15) Mechanic Winches<br>16) Hydraulic Winches	From year:<br>From year:	From year:<br>From year:	From year:<br>From year:
17) Main target species	1)	1)	1)
	2)	2)	2)
	3)	3)	3)
	4)	4)	4)
	5)	5)	5)
18) Main fishing grounds (location and depth range)			

Collect data for the case studies on a map			
19) Types of bottoms exploited<br>Sand, mud, hard bottoms, Posidonia meadows etc			
20) Main fishing season			

**HABITUAL FISHING TRIP**			
	From 40's to 60's	From 60's to 80's	From 80's to present
21) Duration (days)			
22) Average number of hauls x day			
23) Average duration x haul (hours)			
24) Depth range			
25) Mean fishing days x			

year			
26) Problems with the gear	☐ Unusual ☐requent	☐ Unusual ☐requent	☐ Unusual ☐equent
	Description	Description	Description
27) Occurrence of fishery with explosives	☐ Unusual ☐requent	☐ Unusual ☐requent	☐ Unusual ☐equent
	Description	Description	Description

CATCH

	From 40's to 60's	From 60's to 80's	From 80's to present
28) Total catch of the past compared with those of the present	☐ Much more abundant (more than three times)	☐ Much more abundant (more than three times)	☐ Much more abundant (more than three times)
	☐ More abundant (two times)	☐ More abundant (two times)	☐ More abundant (two times)
How do you feel?	☐ the same	☐ the same	☐ the same
	☐ Less abundant	☐ Less abundant	☐ Less abundant
	☐ Not able to evaluate	☐ Not able to evaluate	☐ Not able to evaluate
29) Most abundant species	1)	1)	1)
	2)	2)	2)

	3)	3)	3)
	4)	4)	4)
30) Sizes of the target species caught compared with those of the present<br>How do you feel?<br>Provide an example	☐ Larger	☐ Larger	☐ Larger
	☐ More or less the same	☐ More or less the same	☐ More or less the same
	☐ Lower	☐ Lower	☐ Lower
	☐ Not able to evaluate	☐ Not able to evaluate	☐ Not able to evaluate
31) Average number of boxes/kg per fishing day, fishing trip or haul			
32) Range of catch, minimum-maximum (boxes/kg)			
33) Presence of benthic organisms	☐ Much more abundant (more than three times)	☐ Much more abundant (more than three times)	☐ Much more abundant (more than three times)
	☐ More abundant (two times)	☐ More abundant (two times)	☐ More abundant (two times)
	☐ the same	☐ the same	☐ the same
	☐ Less abundant	☐ Less abundant	☐ Less abundant
	☐ Not able to evaluate	☐ Not able to evaluate	☐ Not able to evaluate

Provide examples			
34) Main commercial species discarded	1)	1)	1)
	2)	2)	2)
	3)	3)	3)
	4)	4)	4)
35) Reasons of discarding of commercial species	☐ Not commercial specimens	☐ Not commercial specimens	☐ Not commercial specimens
	☐ Damaged specimens	☐ Damaged specimens	☐ Damaged specimens
	☐ Other	☐ Other	☐ Other
36) Periods with maximum presence of juveniles<br><br>Provide examples			

MARKETING

	From 40's to 60's	From 60's to 80's	From 80's to present
37) More economically relevant species	1)	1)	1)
	2)	2)	2)
	3)	3)	3)
	4)	4)	4)
	5)	5)	5)

SPECIES OF PARTICULAR INTEREST

	From 40's to 60's	From 60's to 80's	From 80's to present
38) Incidental catches<br><br>Cetaceans (dolphins, whales), seals (monk seals, etc…), turtles	☐never<br>☐occasional<br>☐frequent<br>☐very frequent	☐never<br>☐occasional<br>☐frequent<br>☐very frequent	☐never<br>☐occasional<br>☐frequent<br>☐very frequent
	which species:	which species:	which species:

39) Intentional catches (dolphins, seals)	☐never<br>☐occasional<br>☐frequent<br>☐very frequent	☐never<br>☐occasional<br>☐frequent<br>☐very frequent	☐never<br>☐occasional<br>☐frequent<br>☐very frequent
	which species:	which species:	which species:
Gear	☐Harpoon<br>Other	☐Harpoon<br>Other	☐Harpoon<br>Other
Purpose	☐ Sale<br>☐ Private consumption	☐ Sale<br>☐ Private consumption	☐ Sale<br>☐ Private consumption
40) Sighting Frequency<br><br>Cetaceans (dolphins, whales), seals (monk seals, etc…), turtles	☐never<br>☐occasional<br>☐frequent<br>☐very frequent	☐never<br>☐occasional<br>☐frequent<br>☐very frequent	☐never<br>☐occasional<br>☐frequent<br>☐very frequent
	which species:	which species:	which species:
41) Selachian catches in the past compared	☐ Much more abundant (more than three times)	☐ Much more abundant (more than three times)	☐ Much more abundant (more than three times)

with those of the present<br><br>How do you feel?	☐ More abundant (two times)<br>☐ the same<br>☐ Less abundant<br>☐ Not able to evaluate	☐ More abundant (two times)<br>☐ the same<br>☐ Less abundant<br>☐ Not able to evaluate	☐ More abundant (two times)<br>☐ the same<br>☐ Less abundant<br>☐ Not able to evaluate
42) Selachians in terms of percentage on the total catch			
43) Main species caught	1)<br>2)<br>3)<br>4)	1)<br>2)<br>3)<br>4)	1)<br>2)<br>3)<br>4)
44) Species present in the past and now disappeared	1)<br>2)<br>3)<br>4)	1)<br>2)<br>3)<br>4)	1)<br>2)<br>3)<br>4)
45) When do you remember the last catch?			

Notes of the interviewer (new contacts, impressions, etc.)
